# Editorial: Immunomodulation by food components via dendritic cells

**DOI:** 10.3389/fnut.2024.1532493

**Published:** 2024-12-17

**Authors:** Satoshi Hachimura

**Affiliations:** Research Center for Food Safety, Graduate School of Agricultural and Life Sciences, The University of Tokyo, Tokyo, Japan

**Keywords:** dendritic cell, food, lactic acid bacteria, NRF2, mucosal immunity

Recent studies have revealed that various food components and nutrients affect immune responses and may thus have health benefits. These food components or nutrients may enhance immune responses, leading to host defense against infection, or they may inhibit immune responses, suppressing allergy and inflammation. Dendritic cells (DCs; both conventional DCs and plasmacytoid DCs) are important targets in these cases, because they orchestrate a variety of immune responses. The intestinal immune system plays a pivotal role in the induction of immune responses against food. In the case of the T-cell response, intestinal DCs have a greater ability to induce regulatory T cells (Tregs) than do DCs from other sites (1). Therefore, the immune response to food components is likely to be affected by the characteristics of intestinal DCs. There are multiple reports on the effects of food components on intestinal DCs (1–10); however, the regulation of immune responses to food by intestinal DCs has not been fully elucidated. The underlying mechanisms, such as food component recognition, intracellular and intercellular signaling, and the contribution of intestinal microflora and their metabolites, have not been clearly described, so this Research Topic was planned. Two reviews, three original research papers, and one clinical trial concerning this Research Topic have been published.

In the review by Kanauchi et al., the antiviral effects of probiotics are comprehensively discussed in the context of DCs. In the pre-COVID-19 era, several probiotic strains were found to be clinically effective in addressing gastrointestinal infectious diseases, along with the common cold and flu. The review by Kanauchi et al. noted that, in these cases, the bacterial strains have been shown to enhance IgA antibody and natural killer (NK) cell activity via the activation of conventional DCs. The reports on one strain, *Lactococcus lactis* strain Plasma, have been unique in that this strain acts on plasmacytoid DCs, inducing the production of anti-infection factors such as type 1 interferons. In another review, Tezuka and Imai focused on soybean-derived molecules, including isoflavones, saponins, flavonoids, and bioactive peptides, which act directly on mononuclear phagocytes, including DCs, to fine-tune immune responses.

The group of my co-topic editor, Chiharu Nishiyama, has contributed two papers. In their first paper (Kodama et al.), they focused on β-damascone, a major ingredient of rose fragrance. Damascone inhibited the functions of DCs, including the antigen-dependent proliferation of T cells, DC-induced T-helper (Th)1 development, and the toll-like receptor (TLR)-ligand-induced production of inflammatory cytokines by DCs. β-Damascone treatment increased the protein level of the transcription factor NF-E2-related factor 2 (NRF2). Nrf2^−/−^ DCs induced Th1 development and produced IL-12p40, even in the presence of β-damascone, whereas these functions by Nrf2^+/−^ DCs were inhibited by β-damascone under the same conditions. The oral intake of β-damascone intake suppressed ear swelling in contact hypersensitivity (CHS) model mice, but not in CHS-induced Nrf2^−/−^ mice. These results indicate that this rose aroma compound suppresses DC-mediated immune responses by activating the NRF2 pathway in DCs and therefore has potential for preventing or attenuating immune-mediated diseases.

In a second study by Prof. Nishiyama's group, they showed that a gut lactic acid bacterial metabolite, 10-oxo-cis-6, trans-11-octadecadienoic acid (γKetoC) inhibited the release of inflammatory cytokines from BMDCs (bone-marrow-derived DCs) and splenic DCs through stimulation of the NRF2 pathway (Ando et al.). Various gut bacteria possess enzymes that produce hydroxy fatty acids (FAs), oxo FAs, conjugated FAs, and partially saturated FAs as secondary metabolites from polyunsaturated FAs. Among these derivatives, Prof. Nishiyama's group identified γKetoC, a γ-linolenic acid-derived enon FA, as an effective immunomodulator that inhibited the antigen-induced immunoactivation and lipopolysaccharide-induced production of inflammatory cytokines. The suppressive effects of γKetoC or of an agonist of GPCRs (G-protein-coupled receptors) 40 and 120 on the release of these cytokines was reduced in Nrf2^−/−^ BMDCs. Furthermore, they showed that oral administration of γKetoC significantly reduced body weight loss and improved colitis in wild-type C57BL/6 and Nrf2^+/−^ mice. In contrast, the pathology of colitis deteriorated in Nrf2^−/−^ mice, even with the administration of γKetoC. These results demonstrated the involvement of the NRF2 pathway and GPCRs in γKetoC-mediated anti-inflammatory responses. These studies by Prof. Nishiyama's group highlight the importance of NRF2 as a mediator in the modulation of DCs by foods and natural substances, including by gut microbiota.

In a third study—our own—we examined the effect of *Lactococcus lactis* subsp. *cremoris* YRC3780—a lactic acid bacterial strain isolated from kefir, a traditional fermented milk product of the Caucasus region—on the intestinal immune responses mediated by intestinal DCs (Nakagawa et al.). It has been shown previously by our group in both animal and human studies that *L. cremoris* YRC3780 ameliorates the signs or symptoms of pollinosis (11, 12). In an atopic-dermatitis-like murine model of skin inflammation, oral administration of this bacterium alleviated allergen-induced dermal responses (13). We elucidated the gene expression of mesenteric lymph node (MLN) DCs, as well as the CD4^+^ T-cell responses induced by these MLN DCs, with particular attention to the induction of regulatory T cells (Tregs). *Lactococcus cremoris* YRC3780 enhanced the expression of genes involved in Treg induction in MLN DCs, such as *Aldh1a2* (encoding retinaldehyde dehydrogenase 2; RALDH2), *Itgav* and *Itgb8* (encoding integrins αv and β8, respectively), and *Il10*. It also induced the production of Foxp3^+^CD4^+^ T cells in an MLN DC and CD4^+^ T-cell co-culture system. The use of MLN DCs enabled us to show that this lactic acid bacterium promoted the induction of Th1 cells and Tregs and suppressed the induction of Th2 cells, thus regulating the balances of Th1–Th2 and Treg–Th17 cells, via antigen presentation by intestinal DCs.

Finally, a clinical trial using a lactic acid bacterium, *Heyndrickxia coagulans* strain SANK70258 (HC), was reported by Aida et al.. They conducted a randomized, double-blind, placebo-controlled, parallel-group study to comprehensively evaluate the effects of HC on immunostimulatory capacity, upper respiratory tract infection (URTI) symptoms, and changes in intestinal organic-acid composition. The results of a questionnaire survey of URTI symptoms showed that runny nose, nasal congestion, sneezing, and sore throat scores, as well as the cumulative number of days of these symptoms, were significantly lower in the HC group than in the placebo group during the study period. The salivary secretory immunoglobulin A (sIgA) concentration was significantly higher, and the NK cell activity tended to be higher, in the HC group than in the placebo group. In addition, Aida et al. performed an exposure culture assay of inactivated influenza virus on peripheral blood mononuclear cells (PBMCs) isolated from the blood of participants in the HC and placebo groups. Analysis of gene expression in the PBMCs after culture completion showed that IFN (interferon)α and TLR7 expression levels were significantly higher in the HC group than in the placebo group. In addition, the expression levels of CD304, one of the surface antigens of plasmacytoid DCs, tended to be higher in the HC group than in the placebo group. The HC group showed a significantly greater increase in the intestinal butyrate concentration than the placebo group. HC intake also significantly suppressed the levels of IL-6 and tumor necrosis factor α produced by PBMCs after exposure to inactivated influenza virus. These results suggested that HC activated plasmacytoid DCs expressing TLR7 and CD304 and strongly induced IFNα production, subsequently activating NK cells and increasing sIgA levels; it also induced anti-inflammatory effects, with increased intestinal butyrate levels. These changes appear to have contributed to the acquisition of host resistance to viral infection and URTI prevention. The results of this clinical trial suggest that the plasmacytoid DC population may be a target for enhancing host immunity by using not only *L. lactis* strain Plasma [(6); Kanauchi et al.] but also other probiotics.

Although the mechanisms underlying the regulation of immune responses to food by DCs have not yet been fully elucidated, the papers presented in our Research Topic clearly demonstrate that DCs—both conventional and plasmacytoid—are important targets of immunomodulation by foods. Further research in this area is awaited, and will contribute to our understanding of immune responses to food components and to the development of functional foods.
